# Development of bone marrow lesions is associated with adverse effects on knee cartilage while resolution is associated with improvement - a potential target for prevention of knee osteoarthritis: a longitudinal study

**DOI:** 10.1186/ar2911

**Published:** 2010-01-19

**Authors:** Miranda L Davies-Tuck, Anita E Wluka, Andrew Forbes, Yuanyuan Wang, Dallas R English, Graham G Giles, Richard O'Sullivan, Flavia M Cicuttini

**Affiliations:** 1 Department of Epidemiology and Preventive Medicine, Monash University, Central and Eastern Clinical School, Alfred Hospital, Melbourne, VIC 3004, Australia; 2 Baker Heart Research Institute, Commercial Road, Melbourne, VIC 3004, Australia; 3 Cancer Epidemiology Centre, The Cancer Council Victoria, Carlton, VIC 3053, Australia; 4 Centre for Molecular, Environmental, Genetic and Analytic Epidemiology, School of Population Health, The University of Melbourne, Carlton, VIC 3053, Australia; 5 MRI Unit, Symbion Imaging, Epworth Hospital, Richmond, VIC 3121, Australia

## Abstract

**Introduction:**

To examine the relationship between development or resolution of bone marrow lesions (BMLs) and knee cartilage properties in a 2 year prospective study of asymptomatic middle-aged adults.

**Methods:**

271 adults recruited from the Melbourne Collaborative Cohort Study, underwent a magnetic resonance imaging scan (MRI) of their dominant knee at baseline and again approximately 2 years later. Cartilage volume, cartilage defects and BMLs were determined at both time points.

**Results:**

Among 234 subjects free of BMLs at baseline, 33 developed BMLs over 2 years. The incidence of BMLs was associated with progression of tibiofemoral cartilage defects (OR 2.63 (95% CI 0.93, 7.44), *P *= 0.07 for medial compartment; OR 3.13 (95% CI 1.01, 9.68), *P *= 0.048 for lateral compartment). Among 37 subjects with BMLs at baseline, 17 resolved. Resolution of BMLs was associated with reduced annual loss of medial tibial cartilage volume (regression coefficient -35.9 (95%CI -65, -6.82), *P *= 0.02) and a trend for reduced progression of medial tibiofemoral cartilage defects (OR 0.2 (95% CI 0.04, 1.09), *P *= 0.06).

**Conclusions:**

In this cohort study of asymptomatic middle-aged adults the development of new BMLs was associated with progressive knee cartilage pathology while resolution of BMLs prevalent at baseline was associated with reduced progression of cartilage pathology. Further work examining the relationship between changes and BML and cartilage may provide another important target for the prevention of knee osteoarthritis.

## Introduction

There is increasing interest in the role of bone marrow lesions (BMLs), detected by magnetic resonance imaging (MRI), in the pathogenesis of knee osteoarthritis (OA) [1,2]. Histological examination of BMLs in knees has reported that they may represent areas of osteonecrosis, oedema, trabecular abnormalities and bony remodeling [3]. BMLs are present in both symptomatic [4-7] and asymptomatic populations [8,9]. Although BMLs are found to be extremely common in OA populations and, once present, are unlikely to resolve [7,10,11], in asymptomatic populations they tend to have a more fluctuating course [12]. BMLs have most commonly been described in relation to mechanical factors such as trauma [13-16], knee malalignment [17], and increased body weight [8]. However, more recently systemic factors such as osteo-protective medications [18] and nutritional factors [19,20] have been reported to affect the risk of BMLs.

Very little is known about the relation between BMLs and other changes in knee structures in asymptomatic, clinically healthy populations. Most previous studies have focussed on symptomatic populations with established knee OA [6,10,11,21], where BMLs are associated with knee symptoms [4,21-25] and progression of structural changes including joint space narrowing [17], loss of cartilage [6,26] and increased prevalence and severity of cartilage defects [23,27]. More recently in an asymptomatic population, the presence of BMLs at baseline was shown to be associated with longitudinal progression of cartilage defects and loss of cartilage volume [28] suggesting that BMLs also have a pathogenic role in pre-clinical OA.

The significance of development or resolution of prevalent BMLs has only recently been examined in populations with, or at high risk of, knee OA [6,7,29]. In two of these studies, the majority of BMLs persisted so both could only examine the effect of change in size of the BMLs, had limited ability to examine incident BMLs, and had no power to investigate resolution [6,7]. In contrast, for participants of the Multi-centre Osteoarthritis Study (MOST) who either had or were at high risk of OA, approximately 40% of BMLs completely resolved and about one-third of cartilage locations developed new BMLs over 30 months, but no significant association between resolution of BMLs and change in cartilage was seen. In addition, the presence, resolution and progression of the BMLs was observed simultaneously within the same knee suggesting that complete resolution of all BMLs in a knee occurred less frequently. Worsening of BMLs and development of new BMLs was associated with increased cartilage loss compared with where BMLs remained stable [29]; however, no comparison between knees with incident BMLs and knees that remained free of BMLs was made. Recently, we have shown for asymptomatic populations that BMLs fluctuate with about 50% resolving and about 14% of people developing new ones over two years [12,30]. Thus, the aim of this study was to examine the relation between incident BMLs and the resolution of BMLs prevalent at baseline and change in knee cartilage over two years in a cohort of asymptomatic middle-aged adults.

## Materials and methods

### Participants

The study was conducted within the Melbourne Collaborative Cohort Study, a prospective cohort study of 41,528 people, assembled to examine the role of lifestyle and genetic factors in the risk of cancer and chronic diseases in Melbourne, Australia [31]. Participants for the current study were recruited from this cohort in 2003-04 if they were aged between 50 and 79 years without any of the following exclusion criteria: a clinical diagnosis of knee OA as defined by American College of Rheumatology criteria [32]; knee pain lasting for more than 24 hours in the past five years; a previous knee injury requiring non-weight bearing treatment for more than 24 hours or surgery (including arthroscopy); a history of any form of arthritis diagnosed by a medical practitioner or a contraindication to MRI, as previously described [33]. The study was approved by The Cancer Council Victoria's Human Research Ethics Committee and the Standing Committee on Ethics in Research Involving Humans of Monash University, Melbourne. All participants gave written informed consent.

### Anthropometric data

Height (cm) was measured using a stadiometer with shoes removed at baseline (1990-94). Weight (kg) was measured with bulky clothing removed at the time of MRI. Body mass index (BMI) was calculated from these data (weight (kg)/height^2 ^(m^2^)).

### MRI and the measurement of BMLs, cartilage volume and defects

#### MRI

An MRI of the dominant knee (defined as the lower limb from which the subject stepped off from when initiating gait) for each participant was performed between October 2003 and December 2004 and approximately two years later, as described on a 1.5-T whole body MR unit (Philips, Medical Systems, Eindhoven, the Netherlands) using a commercial transmit-receive extremity coil [9]. The following sequences and parameters were used: fat suppressed, gradient recall acquisition in the steady state, three dimensional T1-weighted (58 msec/12 msec/55°, repetition time/echo time/flip angle), one signal average, slice thickness 1.5 mm, field of view 16 cm and matrix 512 × 512 scans. In addition, a coronal T2-weighted fat-saturated acquisition, (3500 to 3800 msec/20/80 msec/90°, repetition time/echo time/flip angle), two signal averages, echo train length of 10, with a slice thickness of 3.0 mm, a 1.0 inter slice gap, 1 excitation, a field of view of 13 cm, and a matrix of 256 × 192 pixels was also obtained [8].

#### Assessment of BMLs

BMLs were defined as areas of ill-defined increased signal intensity adjacent to subcortical bone present in either the medial or lateral, distal femur or proximal tibia assessed of coronal T2-weighted fat-saturated images [34]. Two trained observers (MD and AW), who were blinded to patient characteristics, as well as sequence of images, together assessed the presence of lesions for each subject. The presence or absence of a BML was determined as previously described [34]. Two trained observers, who were blinded to patient characteristics, as well as sequence of images, together assessed the presence of lesions for each subject. The presence or absence of a BML was determined as previously described [17,28]. Briefly, a lesion was defined as present if it appeared on two or more adjacent slices underlying the cartilage plate. A BML was defined as 'incident' if it was present at follow up in the knees without BMLs at baseline. A BML was defined as 'resolved' if it was present at baseline but disappeared at follow up. A BML was classified as 'persistent' if it was present in the same location on both the baseline and follow-up scans. The reproducibility for determination of the BMLs was assessed using 60 randomly selected knee MRIs (κ value 0.88, *P *< 0.001).

#### Measurement of cartilage volume

The volumes of individual cartilage plates (medial and lateral tibia) were measured from the total volume by manually drawing disarticulation contours around the cartilage boundaries on each section on a workstation as described [33]. The coefficients of variation for the medial and lateral tibial cartilage volume measures were 3.4% and 2.0% respectively [35,36]. Annual change in cartilage volume was calculated as follow up cartilage volume subtracted from initial cartilage volume then divided by the period of time between MRI scans, as described [35].

#### Assessment of cartilage defects

Cartilage defects were graded on the sagittal T1-weighted MR images with a classification system as previously described [37-39], in the medial and lateral tibial and femoral cartilages. Cartilage defects were graded as follows: grade 0, normal cartilage; grade 1, focal blistering and intracartilaginous low-signal intensity area with an intact surface and bottom; grade 2, irregularities on the surface or bottom and loss of thickness of less than 50%; grade 3, deep ulceration with loss of thickness of more than 50%; grade 4, full-thickness cartilage wear with exposure of subchondral bone. A cartilage defect also had to be present in at least two consecutive slices. The baseline and follow-up cartilage defects were graded in duplicate (the cartilage defects were re-graded one month later), unpaired and blinded to the sequence. The defect scores at medial tibiofemoral (0-8) and lateral tibiofemoral (0-8) compartments were used in the study. Intra-observer reliability (expressed as intraclass correlation coefficient, ICC) was 0.90 for the medial tibiofemoral compartment and 0.89 for the lateral tibiofemoral compartment [40]. Change in cartilage defects in a compartment was classified as to whether or not they progressed (i.e. increase in cartilage defect score), regressed (i.e. reduction in cartilage defect score) or remained stable (i.e. no change in cartilage defect score).

### Statistical analysis

All variables were assessed for normality by visually inspecting histograms. Baseline characteristics for the 271 subjects who completed both MRI scans were tabulated. Linear regression was used to examine the compartment specific relation between having an incident or resolved BML and annual change in cartilage volume. Logistic regression was used to determine the compartment specific odds of cartilage defect progression versus regression/stability in relation to if a person had an incident BML or a resolved BML over two years. Potential confounders of age, gender, BMI, and tibial plateau area for annual change in cartilage volume were included in multivariate analyses. A *P *value less than 0.05 (two-tailed) was regarded as statistically significant. All analyses were performed using the SPSS statistical package (version 15.0.0, SPSS, Cary, NC, USA).

## Results

Two hundred and seventy-one (90%) of the originally recruited 297 participants completed both MRI scans at baseline and approximately two years later. Reasons for loss to follow up included: death (3), withdrawal for health reasons (4), withdrawal of consent (10), ineligible for follow up (pacemakers) (4), and inability to be contacted (5). The only significant difference between those who completed follow up and those who were lost to follow up was that those lost to follow up were slightly heavier (*P *= 0.01). Of the 271 participants, 234 (86%) did not have a BML in their knee at baseline. Over the two-year study period, 33 (14%) developed a BML in their knee. Of the 37 (14%) participants who had a BML in their knee at baseline, 20 (54%) persisted and 17 (46%) resolved over the two-year study period. The characteristics of the participants are presented in Table 1.

**Table 1:** Characteristics of participants

	**With BMLs at baseline** **(n = 37)**			**Free of BMLs at baseline** **(n = 234)**		
	
	**BMLs persisted** **(n = 20)**	**BMLs resolved** **(n = 17)**	***P *value**	**BMLs developed** **(n = 33)**	**No BMLs developed** **(n = 201)**	***P *value**
	
*Age (years)*	58.6 (5.5)	57.8 (6.4)	0.70^1^	57.7 (5.9)	57.8 (5.0)	0.90^1^
*Gender (% female)*	13 (65%)	11 (65%)	0.98^2^	23 (70%)	122 (61%)	0.30^2^
*Body mass index (kg/m*^2^)	25.9 (3.9)	24.8 (4.1)	0.50^1^	28.0 (5.1)	25.4 (3.7)	0.01^1^
*Annual change in cartilage volume (μl)*						
*Medial tibial*	36.0 (39.2)	10.5 (45.9)	0.13^1^	34.0 (54.6)	19.5 (50.0)	0.08^1^
*Lateral tibial*	25.6 (67.2)	28.4 (42.1)	0.08^1^	37.6 (57.0)	21.0 (48.4)	0.88^1^
*Progression of tibiofemoral cartilage defects, number (%)*						
*Medial*	7 (35%)	3 (18%)	0.15^2^	11 (33%)	44 (21%)	0.24^2^
*Lateral*	9 (18%)	8 (47%)	0.008^2^	15 (45%)	47 (23%)	0.90^2^

Mean (standard deviation) unless otherwise stated. BML = bone marrow lesion.

^1 ^Independent samples t-test

^2 ^Chi-squared test

### Relation between incident BMLs and tibiofemoral cartilage properties

**Figure 1:**
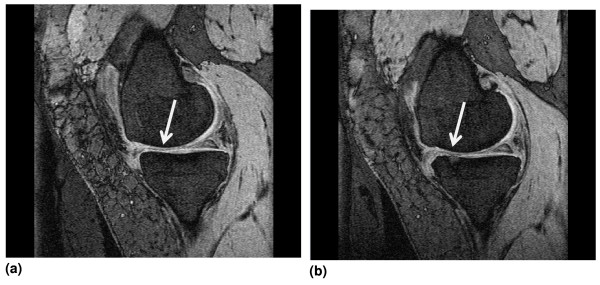
**Magnetic resonance images**. **(a) **Magnetic resonance image of knee showing no bone marrow lesion (BML) and a grade 2 medial tibial defect at baseline. **(b) **Magnetic resonance image showing an incident medial tibial BML and a grade 3 medial tibial defect above the BML at follow up.

The associations between developing an incident BML and annual change in cartilage volume and progression of tibiofemoral cartilage defects are presented in Table 2. Within the medial compartment developing an incident BML was not associated with annual change in medial cartilage volume, but a trend for incidence of medial BMLs being associated with progression of medial tibiofemoral cartilage defects was observed (odds ratio (OR) = 2.63, 95% confidence interval (CI) = 0.93 to 7.44, *P *= 0.07). A similar finding was seen in the lateral compartment. Although incidence of lateral BMLs was not associated with annual change in lateral cartilage volume, having an incident lateral BML was associated with a 3.13 fold (95% CI = 1.01 to 9.68, *P *= 0.05) increased odds of having lateral tibiofemoral defects progress. Figure 1 shows MRI images of knee that developed an incident BML over the two-year period and the worsening of a tibial defect located above the incident BML.

**Table 2:** Relation between compartment specific incident bone marrow lesions and longitudinal change in knee cartilage (n = 234)

	Univariate analysis regression coefficient/odds ratio(95% CI)	*P *value	Multivariate analysis regression coefficient/odds ratio (95% CI)*	*P *value
**Medial compartment**				
*Annual change in cartilage volume*	4.12 (-19.30, 27.60)	0.73	2.37 (-21.78, 26.53)^1^	0.85
*Cartilage defects progress vs no change*	1.86 (0.70, 4.93)	0.21	2.63 (0.93, 7.44)^2^	0.07
**Lateral compartment**				
*Annual change in cartilage volume*	21.2 (-5.86, 48.20)	0.12	18.04 (-9.72, 45.80)^1^	0.2
*Cartilage defects progress vs no change*	3.0 (1.01, 8.93)	0.05	3.13 (1.01, 9.68)^2^	0.05

^1 ^Annual change in tibial cartilage volume if an incident bone marrow lesion (BML) developed compared with if no BML developed after adjusting for age, gender, body mass index (BMI) and respective baseline tibial plateau area

^2 ^Odds ratio for cartilage defects to progress if an incident BML developed compared with if no BML developed after adjusting for age, gender, BMI and respective baseline cartilage volume

CI = confidence interval.

### Relation between resolved BMLs and tibiofemoral cartilage properties

The compartment specific associations between having a BML resolve compared with it persisting over two years and annual change in cartilage volume and progression of tibiofemoral defects are presented in Table 3. Having a medial BML resolve over the study period was associated with a trend for reduced annual loss in medial tibial cartilage volume (regression coefficient = -28.7 μl, 95% CI = -58.11 to 0.68, *P *= 0.05) in univariate analyses. After adjusting for potential confounders this relation became significant (regression coefficient = -35.9 μl, 95% CI = -65 to -6.82, *P *= 0.02). A trend for resolution of medial BMLs and reduced likelihood of progression of medial tibiofemoral defects was also observed in both univariate (OR = 0.23, 95% CI = 0.05 to 1.08, *P *= 0.06) and multivariate analyses (OR = 0.2, 95% CI = 0.04 to 1.09, *P *= 0.06). No relation between the resolution of lateral BMLs and annual change in lateral cartilage volume or progression of lateral tibiofemoral defects was seen.

**Table 3:** Relation between compartment specific resolution compared with persistence of bone marrow lesions and change in knee cartilage (n = 37)

	Univariate analysis regression coefficient/odds ratio (95% CI)	*P *value	Multivariate analysis regression coefficient/odds ratio (95% CI)*	*P *value
**Medial compartment**				
*Annual change in cartilage volume*	-28.70 (-58.11, 0.68)	0.05	-35.90 (-65.00, -6.82)^1^	0.02
*Cartilage defects progress vs no change*	0.23 (0.05, 1.08)	0.06	0.20 (0.04, 1.09)^2^	0.06
**Lateral compartment**				
*Annual change in cartilage volume*	24.70 (-18.88, 68.37)	0.26	23.41 (-23.13, 70)^1^	0.31
*Cartilage defects progress vs no change*	1.08 (0.24, 4.90)	0.92	1.08 (0.22, 5.39)^2^	0.92

^1 ^Annual change in tibial cartilage volume if a bone marrow lesion (BML) resolved vs persisted after adjusting for age, gender, body mass index (BMI) and respective baseline tibial plateau area

^2 ^Odds ratio for cartilage defects to progress if a BML resolved vs persisted after adjusting for age, gender, BMI and baseline cartilage volume

CI = confidence interval.

## Discussion

In this cohort of asymptomatic middle-aged adults, the development of new BMLs in knees free of BMLs at baseline was associated with the progression of tibiofemoral cartilage defects over two years. In contrast, the resolution of BMLs was associated with reduced loss of medial tibial cartilage volume and a trend towards reduced progression of tibiofemoral cartilage defects.

The relation between incident BMLs and change in cartilage has only recently been examined [29]. Among elderly participants with or at high risk of knee OA, development of new BMLs was associated with a worsening cartilage score as assessed using the WORMS (Whole organ MRI score) scale compared with knees where a BML remained stable; however, a comparison of cartilage loss with knees that remained BML free was not performed [29]. Although we did not show a relation between incident BMLs and change in cartilage volume, there was progression of cartilage defects. This may be due to the relative short duration of two years of follow up; in a pain-free population, people are likely to have slower cartilage loss, and also due to the fact that cartilage defects are an earlier and independent marker of cartilage pathology [37]. We have shown that cartilage defects are present in asymptomatic people with no clinical or radiological OA and to be predictors of cartilage loss in healthy people [41] and those with OA [37], independent of initial cartilage volume. Thus, it may be that the relation we have observed between incident BMLs and cartilage defects reflects early cartilage pathology and longer duration of follow up may be needed in order to observe subsequent cartilage volume loss.

In this asymptomatic population we found that the resolution of BMLs over two years was associated with beneficial effects on cartilage as evidenced by reduced loss of tibial cartilage volume and a trend towards reduced progression of tibiofemoral cartilage defects, suggesting this is not simply due to cartilage swelling. Our results are supported by recent observations in OA populations [6,7,29]. For subjects with OA, an increase in size of BML was shown to be associated with increasing C-terminal cross-linking telopeptide of collagen type II levels [6] and increased cartilage loss [7,29]. To our knowledge only one study, the MOST, has examined cartilage changes in knees where BMLs resolved; however, no significant association was observed between resolution of BMLs and change in cartilage [29]. This may, at least in part, be due to the mixed nature of the MOST population because the purpose of the MOST was to examine a population at high risk of OA. In the MOST population, approximately 12% had symptomatic OA, approximately 24% had symptoms and about one-third had a Kellgren Lawrence score greater than or equal to two and past injury and surgery were not excluded. Therefore, the joints of these participants may already be further along the pathological pathway of structural change from the normal joint to one with OA, where the factors culminating in a BML, and acting on the whole knee, are established. In this situation, the reduction in change of cartilage associated with the resolution of BMLs may be lessened. In contrast, our population was asymptomatic and participants with prior injury or knee surgery were excluded.

There is growing evidence to suggest that BMLs have an important role in the pathogenesis of knee OA. They are common and persistent in symptomatic OA where they are associated with pain and progression of OA [4,6,17,21-26]. Although less common in asymptomatic people, BMLs are also associated with progressive knee cartilage pathology [28,42]. In this asymptomatic population with no clinical OA, the development of new BMLs was associated with adverse effects on knee cartilage, while resolution of BMLs was associated with improvement in cartilage. Although it has been suggested that BMLs are largely due to adverse biomechanical factors, we, and other investigators, have shown that systemic factors also affect the risk of BMLs [18,20,43]. It may be that in the observed relation between BMLs and cartilage, factors contributing to the development of BMLs have resulted in impairment of the supply of nutrients and oxygen to the overlying cartilage plate, which may also reduce the strength of the bony support of articular cartilage [44,45]. Our data also suggest that this is reversible because resolution of BMLs was associated with reduction in cartilage defects and cartilage loss. Thus identifying factors that reduce the incidence of BMLs and increase their resolution may offer therapeutic targets in the prevention of knee OA.

This study has a number of potential limitations. Firstly, it examined a healthy asymptomatic population selected on the criteria of no knee pain or injury and therefore, the results are not generalisable to symptomatic populations or people who have injured their knees. On the other hand, the findings from our study can be generalised to populations that may be targeted for primary prevention or early treatment of knee OA. Second, we did not obtain radiographs of the knees, so some subjects may have had asymptomatic radiographic OA. However, we used the American College of Rheumatology clinical criteria of OA [32] to determine the status of knees, and individuals with significant knee injury in the past, pain at baseline, knee surgery or medical diagnosis of any other type of arthritis were excluded. Due to the small number of persistent BMLs we were unable to examine change in BML size. The small number of BMLs may have also reduced our power to detect significant associations and may explain the trends reported. In this study we did not assess knee alignment, which has been shown to be associated with the presence of BMLs [17]. If malalignment were to be a major determinant of BMLs, we would not expect it to change significantly in a healthy asymptomatic population over a period of only two years, so would expect it to underestimate the relations we observed.

## Conclusions

In this cohort study of asymptomatic middle-aged adults the development of new BMLs was associated with progressive knee cartilage pathology, while resolution of BMLs prevalent at baseline was associated with reduced progression of cartilage pathology. Further work examining the relation between changes and BML and cartilage may provide another important target for the prevention of knee OA.

## Abbreviations

BMI: body mass index; BML: bone marrow lesion; CI: confidence interval; CTX-II: C-terminal crosslinking telopeptide of collagen type II; MOST: Multi-centre Osteoarthritis Study; MRI: magnetic resonance imaging; OA: osteoarthritis; OR: odds ratio.

## Competing interests

The authors declare that they have no competing interests.

## Authors' contributions

FC, AW, DE, GG and RO were all involved in the design and implementation of the study including data collection and measurement. MD, AE, AF, YY and FC were involved in the analysis and interpretation of the data. All authors were involved in the manuscript preparation.
